# Get out of my head: social evaluative brain states carry over into post-feedback rest and influence remembering how others view us

**DOI:** 10.1093/cercor/bhae280

**Published:** 2024-07-16

**Authors:** Sasha C Brietzke, Klara Barbarossa, Meghan L Meyer

**Affiliations:** Department of Psychology, Columbia University, New York, NY, United States; Department of Psychological and Brain Sciences, Dartmouth College, Hanover, NH, United States; Department of Psychology, Columbia University, New York, NY, United States

**Keywords:** social cognition, consolidation, default mode network, self, memory, social feedback

## Abstract

Learning how others perceive us helps us tune our behavior to form adaptive relationships. But which perceptions stick with us? And when in the learning process are they codified in memory? We leveraged a popular television series—*The Office*—to answer these questions. Prior to their functional magnetic resonance imaging (fMRI) session, viewers of *The Office* reported which characters they identified with, as well as which characters they perceived another person (i.e. counterpart) was similar to. During their fMRI scan, participants found out which characters other people thought they and the counterpart were like, and also completed rest scans. Participants remembered more feedback inconsistent with their self-views (vs. views of the counterpart). Although neural activity while encoding self-inconsistent feedback did not meaningfully predict memory, returning to the inconsistent self feedback during subsequent rest did. During rest, participants reinstated neural patterns engaged while receiving self-inconsistent feedback in the dorsomedial prefrontal cortex (DMPFC). DMPFC reinstatement also quadratically predicted self-inconsistent memory, with too few or too many reinstatements compromising memory performance. Processing social feedback during rest may impact how we remember and integrate the feedback, especially when it contradicts our self-views.

## Introduction

In the everyday theater of social life, our self-expression is both an act we perform and a play that others critique (Goffman 1959). Driven by a fundamental need to belong (Baumeister and Leary 1995; Leary and Kelly 2009), we hone our “stagecraft” in the pursuit of self-awareness and understanding. This process is facilitated not only by introspection (Wilson and Dunn 2004; Carver 2012), but also by continuous feedback from others (Cooley 1902; Mead 1934; Lundgren 2004). Yet, how we process and later remember how others perceive us, and how that may differ from observing people’s reactions to other actors on the social stage, remains to be fully determined.

Social psychology research consistently demonstrates a bias toward remembering self-relevant (vs. self-irrelevant) information (Symons and Johnson 1997; Sui and Humphreys 2015). However, it remains unclear in the social evaluation context whether this effect may be facilitated by feedback consistent or inconsistent from our self-view. On the one hand, information consistent with our self-view may be folded into our existing schemas more readily than our limited information formed about other people (Bartlett 1950; Taylor and Crocker 1981), therefore abetting memory. On the other hand, information inconsistent from our self-view may invoke feeling misunderstood (Morelli et al. 2014), trigger cognitive dissonance (Festinger 1962; Aronson 1992; Cooper 2019), and/or elicit an emotional response that lingers longer in memory (Ledoux 2000; Levine and Pizarro 2004) than feedback for another person. A third option also remains that both consistent and inconsistent feedback work together equally toward a memory advantage for self over other.

If inconsistent or consistent feedback is better remembered when the feedback is given to the self (versus someone else), what may be the underlying neural mechanisms supporting this phenomenon? A large body of research suggests new information is consolidated in memory during post-encoding rest. In non-human animals, when hippocampal and medial prefrontal patterns engaged during encoding “replay” during post-experience rest, they promote memory (Foster and Wilson, 2006; Hoffman and McNaughton, 2002; Kudrimoti et al. 1999; Jadhav et al. 2016; Kaefer et al. 2020). Although explicit replay is difficult to show in humans, research does suggest humans continue to process encoded information during subsequent rest and that this process makes unique contributions to memory formation. For example, in humans, the hippocampus, as well as other subcortical and cortical regions, show increased functional connectivity during post-encoding rest to facilitate memory (Tambini et al. 2010; Deuker et al. 2013; Tompary et al. 2015; Murty et al. 2017). In fact, brain regions engaged while receiving social feedback—the dorsomedial prefrontal cortex (DMPFC) and anterior insula (AI) (Izuma et al. 2008; Eisenberger et al. 2011; Muscatell 2013; Muscatell et al. 2015, 2016)— also contribute to relevant post-encoding consolidation processes. Increased resting state functional connectivity between a DMPFC functional network and frontoparietal control network after listening to people share personal information predicted memory for their disclosures (Collier and Meyer 2020). Distributed patterns in the AI during the encoding of emotional stimuli are reinstated—or carry over—to influence the processing and memory for neutral items tens of minutes later (Tambini et al. 2017). This area of research suggests committing socioemotional information to memory often does not happen fully in the moment of encoding, and that brain regions associated with encoding socioaffective information (e.g. DMPFC, AI), and memory processes more broadly (e.g. hippocampus) may impact how we remember others’ perceptions of us.

That said, the relationship between post-encoding processes and subsequent memory performance may be more nuanced for social feedback about oneself (vs. others) than the types of stimuli previously studied. Prior research suggests a linear relationship between neural responding during post-encoding rest and subsequent memory (Meyer et al. 2019; Tambini and Davachi 2019; Collier and Meyer 2020). For example, in one study participants watched social and non-social video clips, completed a resting state scan, and a surprise memory test. Participants who most frequently returned to (i.e. reinstated) DMPFC neural patterns engaged while watching social (vs nonsocial) videos, performed preferentially better on the social memory test (Jimenez and Meyer 2024). However, when it comes to finding out how other people perceive you, excessive neural activity during post-encoding rest might be counterproductive. Feedback on the self may spur introspective processes that interfere with memory formation during consolidation phases. People are also prone to ruminate after receiving social feedback (Zoccola et al. 2008). Moreover, engaging the same brain regions associated with receiving social feedback—the DMPFC and AI—during resting state scans is robustly associated with depressive rumination (Makovac et al. 2020; Zhou et al. 2020; Kim et al. 2023). Importantly, rumination can lead to “sticky” mental states that may disrupt memory processes by lingering on irrelevant material (Davis and Nolen-Hoeksema 2000; Altamirano et al. 2010; Zetsche and Joormann 2011). After receiving social feedback on the self, a “mid-range” level of DMPFC and/or AI engagement during post-encoding rest may be optimal to effectively consolidate self-relevant information without succumbing to introspective distractions.

The goals of the present research were to (i) assess whether feedback inconsistent vs. consistent with our self-views is well-remembered compared to our views of others and (ii) explore whether and how this phenomenon plays out during post-encoding rest. Our study tackled these goals by delivering feedback that was either consistent or inconsistent with participants’ own self-views. We recruited viewers of the television show *The Office* to complete a functional magnetic resonance imaging (fMRI) study in which they were led to believe they would learn, on a trial-by-trial basis, which characters from *The Office* other people perceived them to be similar to. In reality, we manipulated their feedback such that half of the trials were inconsistent and half of the trials were consistent with participants’ self-views (i.e. which characters they reported identifying with more prior to the scan). As a control condition for self feedback, participants also observed another supposed participant receive consistent and inconsistent feedback. Resting state scans directly followed the self feedback and other feedback runs, respectively. We were therefore able to characterize the role of consistency in remembering feedback for the self (vs. other), as well as how the brain optimally commits this new information to memory during post-encoding rest.

## Materials and methods

### Participants

Forty-two undergraduates (52.38% women, mean age = 19.98; s.d. = 1.29, racial breakdown: 69.05% White, 19.05% Asian, 7.14% African American, 2.38% Hispanic, and 2.38% who preferred not to answer) were recruited for the present study and screened for any MRI contraindications (e.g. metal in body, claustrophobia, pregnancy). All participants provided informed consent and either received extra credit for a course or a check payment in exchange for their participation. This study was approved by the university’s Institutional Review Board. One participant was removed from analysis due to both excessive head motion and poor memory performance for a total sample size of 41 participants. Deidentified data and accompanying code is made available on OSF: https://osf.io/4a98m/.

### Procedure

**Fig. 1:**
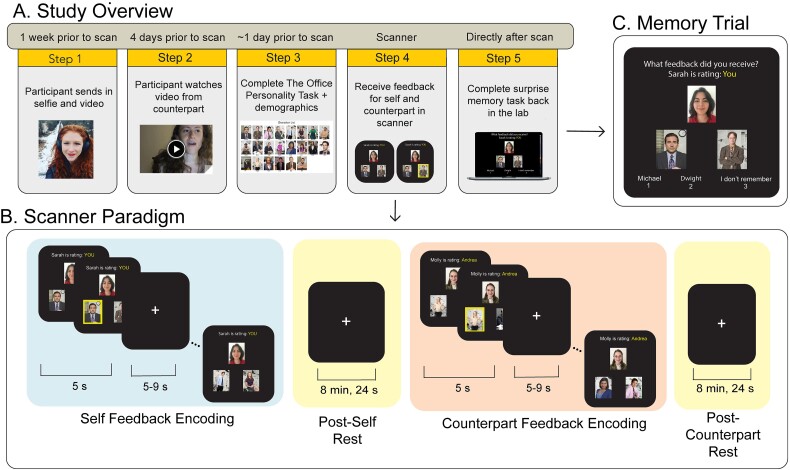
(A) Study overview detailing the timeline of the study. (B) Scanner paradigm. In the scanner, participants received feedback for both themselves (i.e. self feedback encoding) and the participant they saw a video of in step 2 (i.e. counterpart feedback encoding). Each trial featured two components: (i) a photograph of the evaluator, and (ii) the two characters from *TheOffice* the evaluator was choosing between to evaluate the participant. When participants pressed a button, the feedback from the evaluator appeared on the screen by highlighting the chosen character. Each feedback trial was presented for 5 s with a 5–9 s jitter between each trial. Following self feedback encoding and counterpart feedback encoding were resting state scans, each lasting 8 min, 24 s. (C) Memory test. Directly after the scan, participants were brought to a quiet testing room to complete the surprise memory task. Each trial of the memory task was arranged in the same format as the scanner task, except without feedback. Participants were instructed to choose which character they remembered the evaluator choosing for each trial of the task. To help guard against guessing, participants could also select that they did not remember.

*Pre-Scan Experimental Setup.* To ensure participants felt they were both evaluating and being evaluated by real peers, they were told the project was a collaboration with a handful of other universities and participants from the different schools would form impressions of one another. At least one week prior to being scanned, participants sent in a photo and a “get to know you” video of themselves (roughly 1 min in duration) in which they shared basic introductory information about themselves such as their name, class year, major, favorite hobbies, and a fun fact (see Fig. 1A for study overview). They were told their videos would be shared with the other participants at participating schools, who would form impressions of them. In addition to submitting a video and photo of themselves, participants were also instructed that a few days prior to being scanned, they would receive a video of a student at another university—referred to as the “counterpart” hereafter—that they would both watch and evaluate. In reality, the counterpart was an undergraduate participant recruited from another university to submit a video, but who did not actually complete the experiment. All of the counterpart videos were the same across scanned participants with respect to gender identities (i.e. one male-identified and one woman-identified video were selected for the experiment).

After viewing the counterpart’s video prior to the scan, participants were prompted to complete an online survey containing *The Office Personality Task*. The task instructed participants to (i) choose which of two *Office* characters they identified most with (i.e. establishing a self-view) and (ii) choose which of two *Office* characters they felt the counterpart most embodied. All pairwise combinations of 22 *Office* characters were presented for both self and counterpart evaluations. For example, participants indicated whether they identified more with Michael vs. Dwight, Michael vs. Angela, Angela vs. Pam, and so on. The purpose of participants evaluating the counterpart was two-fold: (i) to enhance the believability that participants at other universities indeed rated our participants and (ii) to establish each participant’s “counterpart perception” so that while in the scanner, they could encode the counterpart receive evaluative feedback consistent and inconsistent with the participant’s own perception of them. Setting up the counterpart condition in this way allows us to assess if our findings are relatively specific to a person observing feedback inconsistent vs. consistent with their personal beliefs about themselves versus their personal beliefs about their “non-self” (i.e. counterpart). All participants were screened to ensure they had watched *The Office* prior to enrolling in the experiment.

To examine the potentially confounding effects of valence on feedback consistency (i.e. whether inconsistent feedback is perceived as negative), participants were also prompted to rate their liking for each character from *The Office* on a 0 (not at all) to 100 (extremely) scale.

#### fMRI experiment

In the scanner, participants completed a “self feedback” block (comprising two, 6-minute scans) in which they received feedback on which characters two “evaluators” from other universities reminded them of from *The Office*. Right before receiving their feedback, participants re-watched the video they submitted to jog their memories on what they were evaluated on. Each trial featured two components: (i) a photograph of the evaluator and (ii) the two characters from *The Office* the evaluator was choosing between to evaluate the participant. When participants pressed a button, the feedback from the evaluator appeared on the screen by highlighting the chosen character in yellow (see Fig. 1B for scanner paradigm). Each feedback trial was presented for 5 s with a 5–9 s jitter between each trial.

Participants also completed a “counterpart feedback” block (comprising two, 6-minute scans), in which they observed two different evaluators provide feedback for the counterpart. Similar to the self feedback scans, participants re-watched the counterparts’ video prior to seeing the counterparts’ feedback. Each trial was structurally akin to the self feedback trials, with the caveat being that both the feedback content (e.g. evaluators’ choices) and the evaluators themselves varied.

Evaluators were undergraduates recruited from other universities who submitted a video and photo, but who did not complete the experiment by providing real feedback. Instead, we manipulated the 58 items of self feedback in the scanner such that half of the trials (*n* = 29) were consistent with participant’s self-view (self-consistent) and half of the trials (*n* = 29) were inconsistent (self-inconsistent) with participant’s self-view as assessed from *The Office Personality Task*. Similarly, we manipulated half of the 58 items of counterpart feedback in the scanner so that half of the trials (*n* = 29) were consistent (counterpart-consistent) and half (*n* = 29) were inconsistent (counterpart-inconsistent) with the participant’s counterpart-view. Order of self and counterpart feedback was counterbalanced across subjects.

In between self feedback and counterpart feedback were 8 min, 24 s resting state scans. The length of the resting state scans was chosen to be consistent with prior human memory consolidation studies (Tambini et al. 2010, 2017; Meyer et al. 2019). Including these periods of rest allows us to measure the post-self feedback encoding period, and the post-counterpart feedback encoding period separately to assess which regions are relatively more responsive after receiving self or counterpart evaluative feedback.

#### Surprise memory task

**Fig. 2:**
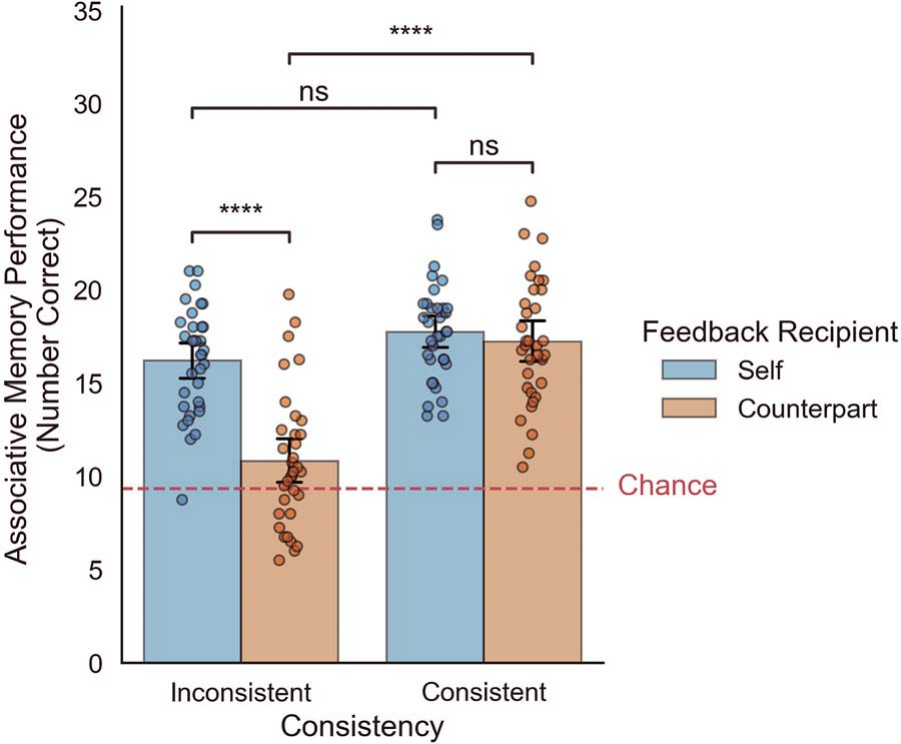
Behavioral memory results demonstrating a target of feedback (self vs. counterpart) by consistency (consistent vs. inconsistent) interaction.

Directly after the scan, participants were brought to a quiet testing room to complete a surprise associative memory task. Each trial of the memory task was arranged in the same format as the scanner task, except without feedback. Participants were instructed to choose which character they remembered the evaluator selecting for each trial of the task (i.e. associate the feedback with the evaluator). To help guard against guessing, participants could also select that they did not remember (Fig. 1C). Presentation of the memory trials occurred within blocks according to the person receiving feedback (i.e. self or counterpart), with the trials themselves randomized within the block. Memory performance was calculated by summing the number of correct evaluator-feedback associations for each participant. Consistent with prior memory consolidation research (e.g. Tambini et al. 2017; Meyer et al. 2019), including the publication whose reinstatement analysis pipeline ours are based on (Schapiro et al. 2018), we removed outliers that were 2 standard deviations below the mean performance per condition—2 for self-consistent memory, 2 for self-inconsistent memory, 3 for counterpart-consistent memory, and 1 for counterpart-inconsistent memory. As depicted in Fig. 2, memory task performance was above chance (>9 remembered items) for every condition.

### fMRI data acquisition

Brain imaging took place on a Siemens Prisma 3 T scanner. Six functional scans—4 feedback task and 2 resting state scans—were acquired using an EPI gradient-echo sequence (2.5 2.5 2.5 mm voxels, TR = 1000 ms, TE = 30 ms, 2.5 mm slice thickness, FOV = 24 cm, matrix = 96 96, flip angle = 59; simultaneous multi-slice (SMS) = 4). A T1-weighted structural image was acquired coplanar with the functional images (0.9 0.9 0.9 mm voxels, TR = 2300 ms, TE = 2.32 ms, 0.9 mm slice thickness, FOV = 24 cm, matrix = 256,256, flip angle = 8). Sequence optimization was derived using optimize design software (https://www.bobspunt.com/easy-optimize-x/). Each feedback run was 5.8 min in duration and each resting state scan was 8.4 minutes in duration.

### fMRI preprocessing

Results included in this manuscript come from preprocessing performed using fMRIPrep 20.2.2 (Esteban et al. 2020, RRID:SCR_016216), which is based on Nipype 1.6.1 (Gorgolewski et al. 2011, RRID:SCR_002502).

For each of the six BOLD scans found per subject (across all tasks and sessions), the following preprocessing was performed. First, a reference volume and its skull-stripped version were generated using a custom methodology of fMRIPrep. BOLD scans were slice-time corrected using 3dTshift from AFNI 20160207 (Cox and Hyde 1997, RRID:SCR_005927). Head-motion parameters with respect to the BOLD reference (transformation matrices, and six corresponding rotation and translation parameters) are estimated before any spatiotemporal filtering using mcflirt (FSL 5.0.9, Jenkinson et al. 2002). Susceptibility distortion correction was omitted. The BOLD reference was then co-registered to the T1w reference using flirt (FSL 5.0.9, Jenkinson and Smith 2001) with the boundary-based registration (Greve and Fischl 2009) cost-function. Co-registration was configured with 9 degrees of freedom to account for distortions remaining in the BOLD reference. The BOLD time-series (including slice-timing correction when applied) were resampled onto their original, native space by applying the transforms to correct for head-motion. Several confounding time-series were calculated based on the preprocessed BOLD: framewise displacement (FD), DVARS (a measurement reflecting the rate of change in successive volumes used to assess image quality), and three region-wise global signals. FD was computed using two formulations following Power (absolute sum of relative motions, Power et al. 2014) and Jenkinson (relative root mean square displacement between affines, Jenkinson et al. 2002). FD and DVARS are calculated for each functional scan, both using their implementations in Nipype (following the definitions by Power et al. 2014). The three global signals are extracted within the CSF, the WM, and the whole-brain masks. The head-motion estimates calculated in the correction step were also placed within the corresponding confounds file. The confound time series derived from head motion estimates and global signals were expanded with the inclusion of temporal derivatives and quadratic terms for each (Satterthwaite et al. 2013). Frames that exceeded a threshold of 0.5 mm FD or 1.5 standardized DVARS were annotated as motion outliers. The BOLD time-series were resampled into standard space, generating a preprocessed BOLD scan in MNI152Nlin2009cAsym space. After preprocessing, we smoothed each scan using a 6 mm full width at half maximum (FWHM) smoothing kernel.

Prior to analyzing the resting state scans, we additionally denoised the preprocessed data by regressing out the six motion parameters, each motion parameter’s derivative and square of the derivative, motion spikes, linear drift, mean CSF value, and global signal.

### Encoding analysis ​​

Encoding effects for self-consistent, self-inconsistent, counterpart-consistent, and counterpart-inconsistent feedback were calculated by modeling the events of interest convolved with the canonical hemodynamic response function in a general linear model. This model included nuisance regressors for the 6 motion parameters (x, y, z directions and roll, pitch, yaw rotations), each motion parameter’s derivative and square of the derivative, linear drift, and scan constants. We additionally regressed out TRs in non-steady state and TRs that exhibited spikes of motion found from global signal outliers and outliers derived from frame differencing (each 3 standard deviations).

### Reinstatement analysis

We employed reinstatement analysis to assess the possibility that neural responses after receiving self-inconsistent feedback codify it in memory (Fig. 4). Reinstatement analysis is a method that counts how many times templates (i.e. multivariate patterns of activity during encoding) are carried over into resting state scans (Schapiro et al. 2018). Using this method, we can assess whether people preferentially return to the state they were in when they encoded inconsistent feedback during post-encoding rest to help commit it to memory. The reinstatement approach was used because inconsistent and consistent conditions are in the same encoding block (i.e. self-consistent and self-inconsistent trials appear in the self-block; counterpart-consistent and counterpart-inconsistent trials appear in the counterpart block). If we instead measured differences in, for example, functional connectivity during post self-encoding rest versus post counterpart-encoding rest, it would be difficult to know if this was due to the connectivity reflecting consolidation of self-inconsistent information specifically. Using the reinstatement approach allowed us to separately assess the extent to which self-inconsistent feedback specifically is more strongly returned to during post feedback rest to promote memory for that information.

In line with the reinstatement analysis pipeline developed by Schapiro et al. (2018), we generated 6 multivariate template patterns using beta images for each condition (i.e. Self [collapsed across consistencies], Counterpart [collapsed across consistencies], Self-Inconsistent, Self-Consistent, Counterpart-Inconsistent, and Counterpart-Consistent) within regions-of-interest (ROIs) identified during first-level contrasts at encoding (see results section for ROI definition).

Our approach to ROI selection is similar to a functional localizer, given that we define our ROI from univariate activity during encoding and then examine how voxels in that ROI behave in the separate rest scan. Although we are applying the multivariate, spatial pattern within that ROI from encoding to subsequent rest, univariate activity and spatial patterns are distinct approaches with different assumptions. Indeed different multivariate spatial patterns can produce the same mean, univariate level of activity within an ROI (Kriegeskorte and Kievit 2013).

To identify reinstatement at each rest period (i.e. Post-Encoding Self Rest and Post-Encoding Counterpart Rest), we correlated each TR during every rest period (nTRs = 504) with each template, resulting in 2 matrices (each 4×504 in size) filled with Fisher-r-to-z transformed correlation values signifying the correspondence between the encoding multivariate template and the multivariate activity at rest at every timepoint. As in Schapiro et al. (2018), a reinstatement was defined as a correlation value >1.5 standard deviations above the mean of all correlations across conditions within each subject. Within each rest period, the number of reinstatements were summed together to represent the amount of reinstatement each participant experienced.

As a robustness check to ensure that our findings were not artifacts of the analysis pipeline, we conducted a control analysis in which we randomly sampled beta values from the encoding scans to create four scrambled templates: self-inconsistent, self-consistent, counterpart-inconsistent, and counterpart-consistent.

## Results

### Behavioral results

*Memory Test.* Directly following the scan, participants completed a surprise, associative memory task to assess which types of social feedback stuck better in memory. In line with prior research (Symons and Johnson 1997; Sui and Humphreys 2015), there was a main effect for the target of feedback such that participants remembered more feedback for the self than for the counterpart (*t*(33) = 7.02, *P* < 0.001, *d* = 1.20; see Fig. 2). Importantly, this main effect was driven by performance on inconsistent trials. There was a significant target (self vs. counterpart) by consistency (consistent vs. inconsistent) interaction (ß(132) = 4.85, *P* < 0.001) which was driven by inconsistent self (vs. counterpart memory). Specifically, follow-up, post-hoc t-tests revealed that the interaction was driven by the fact that self-inconsistent feedback was better remembered than counterpart-inconsistent feedback (*t*(33) = 7.14, *P* < 0.001, *d* = 1.22), despite the fact that self-consistent and counterpart-consistent feedback were similarly remembered (*t*(33) = 0.85, *P* = 0.40, *d* = 0.15). Additionally, counterpart-consistent feedback was significantly better recalled than counterpart-inconsistent feedback (*t*(33) = 5.67, *P* < 0.001, *d* = 0.97, see Fig. 2), despite the fact that self-consistent and self-inconsistent feedback were not significantly different from one another (*t*(33) = 1.83, *P* = 0.07, *d* = 0.31). Overall, the memory results suggest that when it comes to finding out people’s perceptions of others, we are just as likely to remember that information if it is consistent with our own perceptions—regardless of if it is consistent with our *self*-perceptions or perceptions of *others*. In contrast, finding out people’s perceptions of *others* that are inconsistent with our perceptions is easily forgotten. And critically, people’s perceptions of ourselves that are inconsistent with our self-perceptions are preserved in memory. We use the word “preserve”—defined as “to keep safe or intact from decay”—given that memory performance for self-inconsistent feedback was equally well remembered as self- and counterpart consistent feedback, and better remembered than counterpart-inconsistent feedback. In other words, it was not susceptible to the memory decay observed for counterpart-inconsistent memory.

#### Likeability ratings

Prior to their scan date, participants rated how much they liked each character from *The Office* on a scale from 0 (not at all)—100 (extremely). We found a significant valence by consistency interaction (ß(156) = −21.55, *P* < 0.001) such that self-inconsistent feedback was perceived as more negative vs. positive (*t*(39) = 8.91, *P* < 0.001, *d =* 1.41) and self-consistent feedback was perceived as more positive vs. negative (*t*(39) = 12.03, *P* < 0.001, *d* = 1.90) (see Supplementary Fig. 1). The same pattern of results appeared for counterpart feedback: the interaction between valence and consistency is significant (ß(156) = −7.85, *P* < 0.001), with feedback that is inconsistent with the participant’s view of the counterpart is perceived as more negative compared to positive (*t*(39) = 2.66, *P* = 0.01, *d =* 0.42), while feedback consistent with the counterpart is perceived as more positive compared to negative (*t*(39) = 3.67, *P* < 0.001*, d =* 0.58; Note: one participant did not complete this portion of the experiment, reflected by the smaller degrees of freedom reported). Given these findings, below we report follow-up analyses that assess the potential role of valence in our neural results.

### DMPFC and the AI/IFG are associated with encoding self-inconsistent feedback

**Fig. 3:**
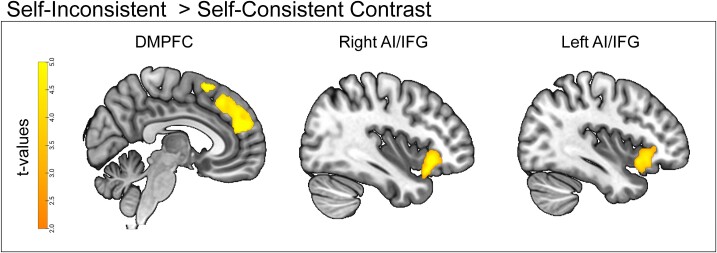
(A) Univariate, encoding contrast maps for self-inconsistent feedback > self-consistent feedback contrast revealing significant activity in DMPFC and bilateral AI/IFG.

Because the behavioral results suggest memory for self-inconsistent feedback was preserved in memory, we next examined the neural correlates for this specific feedback during encoding. Group-level statistical maps were thresholded at *P* < 0.005 and cluster-size thresholds were applied using AFNI’s *3dClustSim* (Cox et al. 2017). A self-inconsistent versus self-consistent contrast revealed significant clusters of activity in DMPFC (*x* = −6, *y* = −16, *z* = 60, *k* = 2238), right AI extending into inferior frontal gyrus (IFG) (*x* = 38, *y* = 26, *z* = −10, *k* = 922), and left AI extending into IFG (*x* = −32, *y* = 18, *z* = −8, *k* = 737; see Fig. 3 and Supplementary Table 1 for full contrast results). The interaction contrast [(Self-Inconsistent—Self-Consistent)—(Counterpart-Inconsistent—Counterpart-Inconsistent)] showed no significant clusters of activity. Because no activity survived cluster correction in the interaction contrast, subsequent reinstatement analyses used the DMPFC, right AI/IFG, and left AI/IFG clusters from the self-inconsistent versus self-consistent contrast as ROIs. We used this contrast to define ROIs, as opposed to the self-inconsistent vs. counterpart-inconsistent contrast, because any neural reinstatement results from the latter would be difficult to interpret. That is, reinstatement results from a self-inconsistent vs. counterpart-inconsistent template could reflect reinstating self-inconsistent patterns or self-patterns more generally.

### Hippocampus is associated with encoding remembered vs. forgotten feedback

In line with prior research implicating the hippocampus in encoding and subsequent memory effects (Kim 2011), a contrast comparing encoded trials that were subsequently remembered vs. forgotten (collapsed across self and counterpart, as well as feedback consistency) revealed a large cluster in the hippocampus and temporal lobe (*x* = 26, *y* = −27, *z* = 2, *k* = 42,468) (see Supplementary Table 2 for a full contrast table). We constrained the ROI to include solely the hippocampus based on a 50 ROI parcellation scheme developed by k-means clustering from the Neurosynth database (Yarkoni et al. 2011; available here: https://identifiers.org/neurovault.image:395092; see Supplementary Fig. 2). We used this hippocampal ROI to perform complementary reinstatement analyses. This allowed us to assess whether any patterns observed in the DMPFC and/or AI also appear in the hippocampus, as well as whether the hippocampus may preferentially reinstate subsequently remembered vs. forgotten feedback (regardless of target and consistency).

### Reinstatement results

**Fig. 4:**
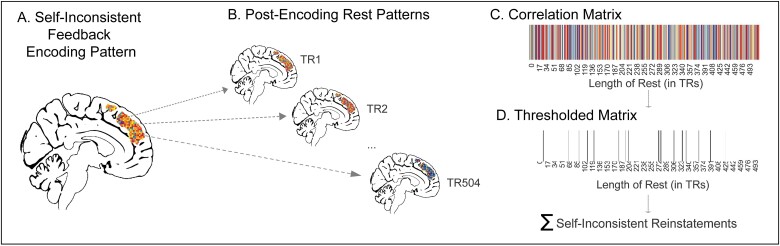
Schematic of reinstatement analysis. This analysis counts the number of times multivariate patterns engaged during encoding (e.g. receiving self-inconsistent feedback) are carried over into subsequent resting state. Within each ROI, we correlated the multivariate template of receiving self-inconsistent feedback (A) with every TR of resting state (B) to generate a correlation matrix (C). Consistent with past reinstatement research (Schapiro et al. 2018), we then thresholded that matrix to any correlation 1.5 standard deviations above the mean and summed the number of TRs exceeding that threshold to generate a quantitative metric of reinstatement (D).

Given that our behavioral results indicate better memory for self (vs. counterpart) inconsistent feedback, we next used neural pattern reinstatement to test the possibility that neural responses after receiving self-inconsistent feedback help explain how it gets preserved in memory. This analysis constructs a multivariate pattern of activity within a predefined ROI (i.e. a template) for each feedback condition during encoding (i.e. self-inconsistent feedback) and correlates this template with every TR of the subsequent resting state scan to identify moments of high correspondence (i.e. a reinstatement; Schapiro et al. 2018; see Fig. 4).

For each of our ROIs of interest (i.e. DMPFC, right AI/IFG, and left AI/IFG), we performed several steps. We (i) examined whether self-inconsistent feedback specifically was reinstated more than counterpart-inconsistent feedback during post-self encoding rest compared to post-counterpart encoding rest and (ii) assessed the relationship between the amount of reinstatement and later memory for that specific feedback type. We further examined whether self feedback (collapsed across consistency) was reinstated more during post-self encoding rest compared to counterpart feedback during post-counterpart encoding rest (see Supplementary Figs 3 and 4). We also explored the potential role of negative valence in our self-inconsistent findings, as well as whether significant results could not be explained by noise by generating “scrambled” templates (see Methods).

### Self-inconsistent feedback is reinstated more in the DMPFC during post-self feedback rest and shows a quadratic relationship with subsequent memory for self-inconsistent items

**Fig. 5:**
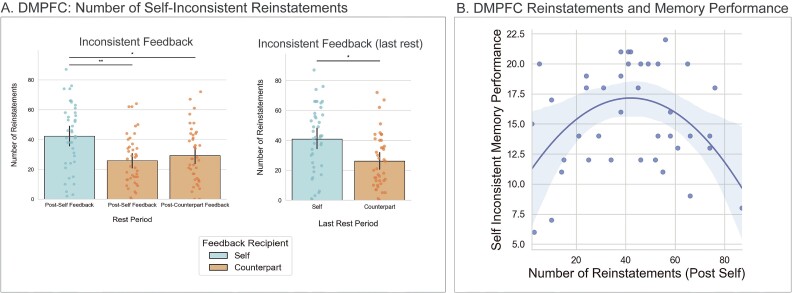
(A) Number of self-inconsistent reinstatements in DMPFC during post-self encoding rest. Inconsistent-self templates were reinstated more than inconsistent-counterpart templates during both post-self encoding rest and post-counterpart encoding rest (left graph). Conversely, during the final rest scan in which all participants had seen all the feedback in the study, self-inconsistent feedback was reinstated more than counterpart-inconsistent feedback (right graph). (B) Plot demonstrating that the number of reinstatements of self-inconsistent feedback within DMPFC during post-self encoding rest displays an inverted U-shaped curved relationship with self-inconsistent items.

We observed multiple findings that collectively suggest DMPFC reinstates self-inconsistent feedback after learning what others think of us. First, the DMPFC self-inconsistent feedback template was reinstated more than the counterpart-inconsistent feedback template during post-self feedback rest (*t*(40) = 3.01, *P* = 0.005, *d* = 0.47; see Fig. 5A). This pattern occurred right after the post-self rest: the same analysis was not significant during post-counterpart feedback rest (*t*(40) = 1.87, *P* = 0.07, *d* = 0.29).

Second, we ruled out alternative explanations that could suggest this observation does not reflect differences in reinstatement, specifically. It is possible that these results are driven by the fact that, for half of our participants, they have not yet encoded counterpart-inconsistent feedback (since the order of self and other feedback encoding was counterbalanced across participants). To rule out this possibility, we performed two follow-up analyses. First, the number of DMPFC reinstatements for self-inconsistent feedback during post-self rest was directly compared to the number of DMPFC reinstatements for counterpart-inconsistent feedback during post-counterpart rest. If DMPFC reinstates all inconsistent feedback equally, then this comparison should be null. However, we again observed that DMPFC reinstatement of self-inconsistent feedback during post-self rest was greater than DMPFC reinstatement of counterpart-inconsistent feedback during post-counterpart feedback rest (*t*(40) = 2.36, *P* = 0.02, *d* = 0.37; see Fig. 5A). Second, we compared the number of DMPFC reinstatements of self-inconsistent feedback and counterpart-inconsistent feedback in each participants’ final rest scan–this ensures that all participants have observed inconsistent feedback for both the self and other while contrasting reinstatements within the same rest period. This analysis was also significant (*t*(40) = 2.55, *P* = 0.02, *d* = 0.40; see Fig. 5A), again suggesting DMPFC reinstates self (vs. counterpart) inconsistent feedback. Overall, these results dovetail with participants’ memory performance on inconsistent trials–we also observed memory performance for self-inconsistent feedback was greater than counterpart-inconsistent feedback.

The parallel analyses with consistent self (vs. counterpart) feedback demonstrated mixed, but generally weak results. There was only a marginal difference in the number of self-consistent (versus counterpart-consistent) reinstatements within DMPFC during post-self feedback rest (*t*(40) = 2.01, *P* = 0.05, *d* = 0.31) and no statistically significant difference during post-counterpart feedback rest (*t*(40) = 1.49, *P* = 0.14, *d* = 0.23). Directly comparing DMPFC reinstatements during the final rest scan also showed that DMPFC reinstatement was marginally different for self-consistent (vs. counterpart-consistent) feedback (*t*(40) = 1.95, *P =* 0.06, *d* = 0.31). These null-to-marginal results are in line with the behavioral results where we also observed that memory performance for consistent self (vs. counterpart) feedback was not meaningfully different.

The DMPFC reinstatement results do not appear to be specific within the context of self feedback: the number of DMPFC self-inconsistent reinstatements was not significantly different from self-consistent reinstatements during post-self rest (*t*(40) = 0.17, *P* = 0.86, *d* = 0.03). This pattern is also similar to our behavioral results, where we observed memory performance was equivalent for self-inconsistent feedback and self-consistent feedback.

We next found support for the possibility that a “mid-range” level of DMPFC engagement during post-encoding rest may be most effective for consolidating self-relevant information without succumbing to introspective distractions. There was a significant quadratic effect between the number of self-inconsistent reinstatements and memory for self-inconsistent feedback (ß(36) = −0.004, *P* = 0.004; see Fig. 5B), which had a lower Bayesian Information Criterion (BIC; 228.5) than the simpler model with just the linear term (233.9), implying the quadratic model fitted the data better. This relationship can be characterized as an inverted U-shaped curve with both higher and lower reinstatements during post-self rest resulting in weaker self-inconsistent memory performance, whereas an intermediate amount of reinstatements corresponds with superior memory (Fig. 5B). There was also a significant quadratic relationship between self-inconsistent reinstatements and self-inconsistent memory during post-counterpart rest (ß(36) = −0.004, *P* = 0.04), with the inclusion of the quadratic term similarly reducing BIC (232.9 compared with 233.9), albeit to a lesser extent than the post self-inconsistent rest models. Further, a post-hoc regression model including both time periods revealed a significant quadratic relationship between self-inconsistent reinstatements and subsequent memory performance for self-inconsistent feedback only for the post-self feedback rest period (ß(36) = −0.003, *P* = 0.04), but not for the post-counterpart feedback rest period (ß(36) = −0.0007, *P* = 0.72). Additionally, the inverted U-shape DMPFC result was not observed in other possible conditions. This relationship was null for the relationship between: (i) the number of self-consistent reinstatements and subsequent self-consistent memory (ß(36) = −0.001, *P =* 0.58), (ii) the number of counterpart-inconsistent reinstatements and subsequent counterpart-inconsistent reinstatements (ß(37) = −0.002, *P =* 0.23), and (iii) the number of counterpart-consistent reinstatements and subsequent counterpart-consistent memory (ß(35) = 0.0005, *P =* 0.68). Overall, the results suggest that the DMPFC plays a preferential and nuanced role in processing and consolidating self-related information that is inconsistent with self-perceptions.

As a robustness check, we randomly sampled beta values from self-inconsistent, self-consistent, counterpart-inconsistent, and counterpart-consistent encoding templates within the DMPFC. After scrambling our labels, we found that self-inconsistent feedback during post-self feedback rest was not reinstated more than counterpart-inconsistent feedback during post-self rest (DMPFC: *t*(40) = −0.24, *P* = 0.81, *d* = −0.04) or counterpart-inconsistent feedback during post-counterpart feedback rest (DMPFC: *t*(40) = 0.16, *P* = 0.87, *d* = 0.03). Moreover, the number of reinstatements and subsequent memory for self-inconsistent feedback in the scrambled analysis did not yield a significant quadratic effect (ß(36) = −0.003, *P* = 0.06 [quadratic]). That said, because the quadratic term was close to significance, we next included both scrambled and unscrambled reinstatement terms in one regression model. In this analysis, the quadratic term for the unscrambled template remained significant (ß(34) = 0.003, *P* = 0.02), while the scrambled template was no longer significant (ß(34) = −0.0007, *P* = 0.67), further indicating the main finding is driven by the unscrambled template. Overall, these follow-up, scrambled analyses indicate the results are not driven by noise.

### Self-inconsistent feedback is reinstated more in the right AI/IFG during post-self feedback rest and positively correlates with subsequent memory for self-inconsistent items

Within the right AI/IFG, we stratified by consistency of feedback to assess whether inconsistent feedback may be reinstated more during post-self feedback rest. Indeed, similar to both the behavioral memory findings and the DMPFC results, we found that the self-inconsistent feedback template was reinstated more than the counterpart-inconsistent feedback template during post-self encoding rest (*t*(40) = 2.32, *P* = 0.03, *d* = 0.36), but not during post-counterpart encoding rest (*t*(40) = 0.87, *P* = 0.39, *d* = 0.14). Conversely, there was no statistical difference in the number of self-consistent reinstatements and counterpart-consistent reinstatements during either post-self encoding rest (*t*(40) = 1.76, *P* = 0.09, *d* = 0.27) or post-counterpart encoding rest (*t*(40) = 1.30, *P* = 0.20, *d* = 0.20).

That said, unlike the DMPFC results, the right AI/IFG results did not survive the follow-up tests of alternative explanations. The number of self-inconsistent reinstatements during post-self rest within right AI/IFG was not greater than the number of counterpart-inconsistent reinstatements during post-counterpart rest (*t*(40) = 1.37, *P* = 0.18, *d* = 0.21), nor was the number of self-inconsistent (vs. counterpart-inconsistent) reinstatements in the final scan (*t*(40) = 1.53, *P* = 0.13, *d* = 0.24). Overall, the right AI/IFG neural reinstatement results complement the DMPFC and behavioral memory results (albeit less robustly): during the rest period following self feedback, there was preferential right AI/IFG reinstatement for self-inconsistent feedback.

Further, the number of self-inconsistent reinstatements during rest after self feedback significantly correlated with self-inconsistent memory accuracy (*r* = 0.33, *P* = 0.04, see Supplementary Fig. 5). This positive correlation for the right AI/IFG was not observed in other conditions. This relationship is null for the relationship between: (i) the number of self-consistent reinstatements and subsequent self-consistent memory (*r* = 0.27, *P* = 0.10), (ii) the number of counterpart-inconsistent reinstatements and subsequent counterpart-inconsistent reinstatements (*r* = 0.16, *P* = 0.32), and (iii) the number of counterpart-consistent reinstatements and subsequent counterpart-consistent memory (*r* = 0.02, *P* = 0.89).

Next we performed control analyses scrambling the betas within each feedback template in the right AI/IFG. After scrambling, our reinstatement comparisons between self-inconsistent and counterpart-inconsistent conditions were null (post-self, self-inconsistent vs. post-self, counterpart-inconsistent: *t*(40) = 1.47, *P* = 0.15, *d =* 0.23; post-self, self-inconsistent vs. post-counterpart, counterpart-inconsistent: *t*(40) = 1.88. *P* = 0.07, *d* = 0.29). Moreover, using the scrambled template, the number of self-inconsistent reinstatements during post self rest did not significantly correlate with self-inconsistent memory accuracy (*r* = −0.10, *P* = 0.54). These follow-up, scrambled analyses indicate the results are not driven by noise.

### Reinstatement of self-inconsistent feedback for correctly recalled items does not meaningfully occur in the left AI/IFG or the hippocampus

Within the left AI/IFG, there was no significant difference in the number of self-inconsistent feedback reinstatements during either post-self feedback rest (*t*(40) = −0.39, *P* = 0.70, *d* = −0.06) or post-counterpart feedback rest (*t*(40) = 0.17, *P* = 0.87, *d* = 0.03). Moreover, there was no significant difference in the number of self-consistent reinstatements during post-self feedback (*t*(40) = 0.01, *P* = 0.92, *d* = 0.02) or post-counterpart feedback rest (*t*(40) = 0.63, *P* = 0.53, *d* = 0.01). Finally, the number of self-inconsistent reinstatements during post-self rest did not significantly correspond to subsequent memory performance for self-inconsistent feedback (*r* = 0.19, *P* = 0.24).

Finally, we performed the reinstatement analysis within our hippocampus ROI. We found no significant difference in the number of self-inconsistent feedback reinstatements during post-self feedback rest (*t*(40) = 1.52, *P* = 0.14, *d* = 0.238) or during post-counterpart feedback rest (*t*(40) = 1.12, *P* = 0.27, d = 0.18). Similarly, we did not find significant differences in the number of self-consistent feedback relative to counterpart-consistent reinstatements during post-self feedback rest (*t*(40) = 0.74, *P* = 0.47, *d* = 0.12) or post-counterpart feedback rest (*t*(40) = 0.03, *P* = 0.98, *d* = 0.004). Further, we did not find any significant correlation between the amount of self-inconsistent feedback reinstatement during post-self rest and subsequent memory for self-inconsistent feedback (*r* = −.12, *P* = 0.48).

Because the hippocampus ROI was defined by a subsequently remembered>subsequently forgotten univariate contrast, we additionally checked whether correct feedback in the hippocampus (regardless of consistency) was reinstated more than incorrect feedback during post-self feedback and post-counterpart feedback rest. Later remembered feedback was not reinstated more than subsequently forgotten feedback in the hippocampus ROI during either post-self feedback rest (*t*(40) = 1.26, *P* = 0.21, *d* = 0.20) or post-counterpart feedback rest (*t*(40) = 0.97, *P* = 0.34, *d* = 0.15). Parallel to the above results, we did not find a significant correlation between the degree to which participants reinstated subsequently remembered items and later memory performance (*r =* −.05, *P* = 0.76).

### Ruling out the possibility that results are driven by “encoding and subsequent memory” effects

The results so far provide evidence that self-inconsistent feedback is reinstated in the DMPFC, and to a lesser extent right AI/IFG, and that reinstatement in these regions predict subsequent memory for feedback inconsistent with our self-views. We next ran follow-up analyses to help ensure these results are not “epiphenomenal,” specifically a carry-over from more meaningful effects occurring during encoding. Prior research frequently observes “encoding and subsequent memory” effects such that greater univariate activity during encoding predicts subsequent memory performance (Fernández et al. 1998; Paller and Wagner 2002; Kim 2011; Long and Kahana 2015). Do we see this in our data? And if so, to what extent are our reinstatement results attributable to residual encoding effects?

We examined whether mean levels of activation during encoding of self-inconsistent information significantly correlated with subsequent memory for self-inconsistent feedback. We did not find that univariate activity in DMPFC or AI/IFG during encoding linearly predicted memory performance for self-inconsistent feedback (*r*_DMPFC_ = 0.005; *r*_left AI/IFG_ = 0.06; *r*_right AI/IFG_ = 0.19; all *P*’s > 0.05). Similarly, we did not find an inverted-U shaped (i.e. quadratic) relationship between DMPFC or AI/IFG brain activity during encoding and subsequent memory for self-inconsistent feedback (ß(36)_DMPFC_ = −8.15, ß(36)_right AI/IFG_ = −15.48, ß(36)_left AI/IFG_ = −1.25; all *P’s* > 0.05). The amount of neural activity *while* receiving self-inconsistent feedback may not play a key role in committing it to memory and reinstatement results reported above are not likely to be “explained away” by the strength of neural encoding.

In contrast to the null “encoding and subsequent memory” results for the DMPFC and AI/IFG, we did see evidence for these relationships in the hippocampus, specifically when ignoring the consistency and target of the feedback. Mean activation during encoding of subsequently remembered items correlated with subsequent associative memory (collapsed across self and counterpart, as well as feedback consistency; *r* = 0.36, *P* = 0.03; see Supplementary Fig. 6). It is notable that when examining this effect for self and counterpart feedback separately, hippocampal encoding activity significantly predicts subsequent memory for counterpart feedback (*r* = 0.39, *P* = 0.02), but not subsequent memory for self feedback (*r* = 0.26, *P* = 0.10), although the self effect follows the same overall trend. Thus, hippocampal mechanisms while encoding how other people perceive their social environment–broadly construed–may play an important role in committing the information to memory.

### Negative valence of feedback does not drive the brain-behavior results

We next aimed to determine whether our post-encoding rest findings could be attributed solely to the negatively perceived feedback, particularly in light of the association between feedback inconsistency and negative valence. To do so, we re-ran our reinstatement results in DMPFC and right AI/IFG separately for inconsistent, negative feedback templates and inconsistent, positive feedback templates. That is, we made a negative, inconsistent multivariate pattern template that included only inconsistent trials in which the selected target was perceived by the participant as less likable. We also separately made a positive, inconsistent multivariate pattern template that included only trials in which the selected target was perceived by the participant as more likable.

In the DMPFC, there were not more negative than positive neural pattern reinstatements during post self-feedback rest for the self-inconsistent feedback trials (*t*(39) = 1.59, *P* = 0.12, *d* = 0.25). Additionally, the negative pattern reinstatements did not drive our subsequent memory results. Within the DMPFC, the number of inconsistent, negative pattern reinstatements did not quadratically or linearly predict subsequent inconsistent, negative memory (Quadratic: ß(35) = 0.0001, *P* = 0.68; Linear: *r* = 0.22, *P* = 0.18).

Within the right AI/IFG, there were more negative than positive neural pattern reinstatements during post self-feedback rest for the self-inconsistent feedback trials (*t*(39) = 2.10, *P* = 0.04, *d* = 0.33). However, the number of inconsistent, negative right AI/IFG pattern reinstatements did not linearly (*r* = 0.16, *P* = 0.34) or quadratically (ß(35) = −0.0005, *P* = 0.81) impact subsequent inconsistent, negative memory. Overall, these results suggest that the negative valence of self-inconsistent feedback does not significantly drive the brain-behavior relationships observed.

## Discussion

While it is widely accepted that self-relevant information is privileged in memory (Symons and Johnson 1997; Sui and Humphreys 2015), the mechanisms that allow us to learn other people’s views of us remain underspecified. To begin to fill this gap, we investigated whether and how social feedback is committed to memory. We found that feedback inconsistent with our own self-views (vs. our views of others) is preserved in memory and this phenomenon occurs, at least in part, through neural processes during rest occurring after receiving the feedback. Specifically, brain states emerging in the DMPFC and to a lesser extent right AI/IFG while receiving self-inconsistent feedback are carried over into post-encoding rest. Moreover, greater right AI/IFG self-inconsistent reinstatements linearly predict better memory for self-inconsistent feedback, whereas self-inconsistent reinstatements within the DMPFC demonstrate a quadratic relationship with subsequent self-inconsistent memory, indicating reinstatements in these regions are functionally relevant to forming memories about the self.

Perhaps unsurprisingly, the consistency of feedback interacted with how likable participants perceived the characters from *The Office*. For both the self and counterpart, inconsistent feedback was more likely to reflect the less likable of the two characters shown whereas consistent feedback was more likely to reflect the more positive of the two characters shown. Importantly, follow-up analyses indicated that the DMPFC results were not confounded with negative valence. The AI/IFG did show more negative vs. positive self-inconsistent feedback reinstatements, though the valence of the reinstatement pattern did not significantly predict subsequent memory. This again suggests that while the AI/IFG may be particularly sensitive to negative information, valence does not confound the subsequent memory results. Overall, our neural pattern reinstatement results fit with self-discrepancy theory (Higgins 1989), which posits that discrepancies between an individual’s actual self-concept and how others perceive them invoke significant cognitive engagement. Indeed, self-discrepancies (regardless of valence) can trigger extensive cognitive activity, often manifesting as rumination, which can impact psychological and neural outcomes (Dickson et al. 2019; Caselli et al. 2014; Roelofs et al. 2007).

The DMPFC findings suggest a “sweet spot” for self-inconsistent memory such that too little reinstatement and too much reinstatement can be deleterious for memory, while those in the middle of the distribution remember self-inconsistent feedback the best. This type of “Goldilocks principle” may reflect that different kinds of cognitive processing are occurring both at encoding and post-encoding rest on either end of the spectrum. For example, at the lower end of the spectrum, participants might be disinclined to focus on self-inconsistent feedback, possibly due to a motivational bias to forget such information. This behavior could lead to reduced reinstatement during post-encoding rest periods. Prior research has linked DMPFC activity with efforts to inhibit the encoding of both cognitive and emotional content, which supports the notion of intentional forgetting (Wylie et al. 2007; Nowicka et al. 2011; Anderson and Hanslmayr 2014). Conversely, at the higher end of the spectrum, participants may be engaging in rumination processes that interfere with accurate consolidation and subsequently result in poorer memory performance. Rumination—perseverative cognition about an event—frequently occurs in response to social experiences, including social evaluation (Zoccola and Dickerson 2012) and has been linked to overgeneralization of autobiographical memories that may limit the retrieval of specific episodes (Watkins and Teasdale 2001; Sutherland and Bryant 2007; Williams et al. 2007). The DMPFC is reliably associated with both rumination (Zhou et al. 2020; Kim et al. 2023) and mental state inference (Van Overwalle 2009; Lieberman et al. 2019). In fact, prior work implicating the DMPFC in social evaluation suggests this region is involved when people ruminate on why another person excluded them (i.e. their intention; Meyer et al. 2015). It is possible that excessive DMPFC reinstatement in our paradigm reflects a focus on why the peer evaluator made their decisions, rather than specifically on the decision itself, ultimately interfering with memory formation.

However, two limitations constrain this interpretation. First, participants’ subjective experience of rumination in response to this paradigm was not measured, making it hard to know if greater DMPFC reinstatements link directly to the conscious experience of rumination. Second, whether neural pattern reinstatement can reflect a conscious percept, such as a ruminative thought, remains to be determined. As such, any interpretation regarding the impact of rumination on memory performance based on neural activity alone remains speculative. Future research could incorporate rumination questionnaires immediately after feedback reception to measure how much participants are subjectively aware of ruminating about the feedback. Another possibility would be to incorporate experience sampling throughout the rest scan. By probing participants’ thoughts across the 8-minute scan, researchers could determine more precisely whether and when rumination-related reinstatement occurs, thus providing a dynamic view of how feedback is reinstated over time during rest and the extent to which participants may be consciously aware of their reinstatement. These approaches would provide a more concrete assessment of the relationship between rumination and neural pattern reinstatement.

The AI/IFG also demonstrated self-inconsistent reinstatement, though unlike the DMPFC’s quadratic relationship with subsequent memory, the AI/IFG showed a linear relationship with subsequent memory. These different patterns may reflect the different information represented by these regions in response to social evaluation. As noted above, the DMPFC may represent the social feedback and the attempt to understand the intention behind the evaluator (e.g. why did they see me this way?). In contrast, the AI/IFG may reflect arousal in response to inconsistent self-feedback, which may be more unexpected and/or surprising. Indeed, the AI is broadly involved in error detection and unexpectedness (Dosenbach et al. 2006; Ham et al. 2013; Klein et al. 2013; Ferdinand and Opitz 2014) and in the social evaluation context has been suggested to represent the autonomic arousal that arises in response to either unexpected and/or negative feedback (Eisenberger and Cole 2012). In terms of post-encoding responses, previous studies found that AI and IFG connectivity carries over from emotional contexts (i.e. viewing distressing scenes) to neutral contexts and influences memory performance (Tambini et al. 2017; Kark and Kensinger 2019; Visser et al. 2022). This prior work used stimuli that are not only negatively valenced, but also high on the arousal dimension. Similarly, we observed greater AI/IFG reinstatement of negative, inconsistent self-feedback than positive, inconsistent self-feedback, though valence on its own did not drive the linear relationship between AI/IFG reinstatement and subsequent memory. We speculate that this is because the AI/IFG more generally represents the arousal that emerges in response to unexpected events and that it is the arousal that linearly facilitates subsequent memory. This fits with prior work showing that high arousal stimuli is preferentially consolidated during sleep (Cunningham et al. 2014)–a key form of rest and a time when systems level consolidation occurs (Walker and Stickgold 2004; Stickgold 2005).

In line with prior literature on “encoding and subsequent memory” effects (Fernández et al. 1998; Paller and Wagner 2002; Long and Kahana 2015), we found that the strength of activity in the hippocampus during encoding predicted subsequent memory for correct items overall, regardless of who received the feedback and the feedback’s consistency. This also fits with work indicating the hippocampus is involved in making decisions about how to interact with people based on our knowledge about them (FeldmanHall et al. 2021). That said, we did not observe meaningful evidence that hippocampal reinstatement occurred and subsequently predicted memory performance. Conversely, activity in right AI/IFG during post-encoding rest—but not during encoding—linearly predicted subsequent memory for self-inconsistent items. One possibility for these findings is that the recruitment of AI/IFG during extended periods of post-encoding rest strengthens the chance of social learning from inconsistent feedback that would otherwise be weaker without the opportunity to rest (Meyer 2019; Meyer et al. 2019). In fact, recent evidence from a less social task (i.e. learning shapes) found that weakly learned items are prioritized during post-encoding rest (Schapiro et al. 2018). Future studies could include self feedback encoding blocks with and without subsequent rest periods to identify whether extended rest is necessary to learn inconsistent social information and if without it, the self-inconsistent feedback results would mirror the counterpart findings.

Another reason why we did not observe hippocampal reinstatement may have to do with the type of cognition needed to commit the feedback to memory. Although the reinstatement during rest approach is inspired by rodent work on hippocampal replay, it is quantitatively different in important ways. Most specifically, replay analysis records from specific neurons and is able to show that the temporal sequence of neural firing during encoding is often literally replayed in sequence during subsequent rest (Pfeiffer 2020). In contrast, reinstatement analysis more simply assesses whether the multivariate pattern activated during encoding is generally returned to, without knowledge of the sequence of neural firing, in later rest. While replay and reinstatement phenomena are similar in that they implicate neural responding during post-encoding rest in memory formation, they may differ in “how” they contribute to memory consolidation during rest. Hippocampal replay may be more episodic in nature–reflecting the precise time, place, and relationships between objects observed during encoding. In contrast, the DMPFC and AI/IFG pattern reinstatement observed here may reflect a more general, semantic representation of the feedback experience, including why the evaluator perceived oneself in such a way (DMPFC) and the corresponding arousal that is experienced (AI/IFG). Indeed, our templates are condition-level templates rather than item-level templates. This further suggests that participants’ return to general social cognitive processes, rather than episodic and temporally sensitive replay of an instance, contributes to our memory results.

It is noteworthy that we did not see evidence of encoding or post-encoding neural activity in the medial prefrontal cortex (MPFC), a region associated with self-referential processing (Denny et al. 2012; Elder et al. 2023; Northoff et al. 2006). One possibility is that the processes engaged in response to social feedback, particularly when the feedback is inconsistent with our self-views, are more centered on attempts to understand the evaluator’s perspective, rather than engage in extensive self-reflection. This fits with earlier work showing stronger DMPFC activation when trying to interpret unclear (vs. clear) social behavior (Jenkins and Mitchell 2010). It also fits with research implicating the AI in responding to prediction errors and unexpectedness (Dosenbach et al. 2006; Ham et al. 2013; Klein et al. 2013; Ferdinand and Opitz 2014). Rather than extensively reflecting on themselves, participants may be trying to make sense of why the other person sees them differently than they see themselves. Past work implicating MPFC in self-referential processing typically requires participants to engage in protracted access to their self-schema (Brietzke and Meyer 2021; Courtney and Meyer, 2020; Denny et al. 2012). Future work may examine whether and how DMPFC and AI/IFG responses to social evaluation may, over time, be incorporated into individuals’ stable self-views, possibly within the MPFC.

Our sample consisted of undergraduate college students, who may represent a relatively homogeneous population in terms of age, socioeconomic status, and educational background. While this limits the direct generalizability to broader populations, research on college students often provides valuable insights into developmental and social processes that are present in young adults more generally. Adolescence–which is an age frame captured in college samples (Sawyer et al. 2018)–is a time when individuals are particularly susceptible to peer influence and evaluation (Somerville, 2013; Steinberg and Morris, 2001), in part due to structural and functional changes occurring within the social brain (Blakemore, 2008). Evaluative feedback studies have shown that adolescents internalize peer feedback to a greater degree than adults (Rodman et al. 2017) and negative evaluations have been found to activate regions associated with the social rejection, including AI (Masten et al. 2009; Will et al. 2016). Studying how undergraduates process social feedback may illuminate neural mechanisms present during a relatively vulnerable time in one’s lifespan. Moreover, future developmental social neuroscience studies may help elucidate when in development post-encoding rest plays the most pivotal role in consolidating social feedback.

More broadly, the results may speak to how social evaluation “gets under the skin” to impact health. It is well-established that even relatively minor forms of social evaluative feedback induces the physiological stress response (i.e. activation in the sympathetic-adrenal-medullary [SAM] axis and the hypothalamic–pituitary–adrenal [HPA axis]; Kudielka et al. 2007; Allen et al. 2017) implicated in poor mental and physical health (McEwen 1998; Gianaros and Jennings 2018). It is also thought that perseverative cognition, such as ruminating about social feedback after encoding it, plays a key role in dysregulating the stress-response such that it confers disease risk (Zoccola et al. 2008; Zoccola and Dickerson 2012; Gianferante et al. 2014). Yet, to date, the majority of neuroscience research investigating social evaluation focuses on the moment of evaluation, rather than the post-event processing in which perseverative cognition occurs. For example, the DMPFC, as well as the AI and dACC, show heightened activity during stressful social evaluation (Eisenberger and Cole 2012; Muscatell et al. 2015) and it has been suggested that the AI plays a key role in representing the autonomic arousal that generates the ensuing stress response (Critchley 2005; Craig 2009; Eisenberger and Cole 2012; Muscatell and Eisenberger 2012). Our findings suggest that information relevant to the self that is inconsistent with our self-views may trigger some discomfort, subsequently get tagged during encoding, and be later reinstated to help learn how others perceive us. Future work may investigate whether the post-encoding reinstatement in the DMPFC and/or AI found here may be a basic mechanism linking social evaluation to physiological stress responding.

## Conclusion

In summary, we provide the first evidence that brain states involved in receiving self-inconsistent feedback are carried over into rest to facilitate learning what others think of us. Understanding the discrepancy between our internal sense of self and the impressions we give off through rest may be stressful, but also help us tune our behavior to optimally navigate social life.

## Supplementary Material

Supplementary_Materials_bhae280
