# Supplementary material for: Get out of my head: social evaluative brain states carry over into post-feedback rest and influence remembering how others view us
Source: Cereb Cortex. 2024 Jul 16;34(7):bhae280. doi: 10.1093/cercor/bhae280 (PMC11250231; doi:10.1093/cercor/bhae280)

## Supplementary Materials

*Supplementary Table 1. Significant clusters found for the Self-Inconsistent>Self-Consistent univariate contrast map.*


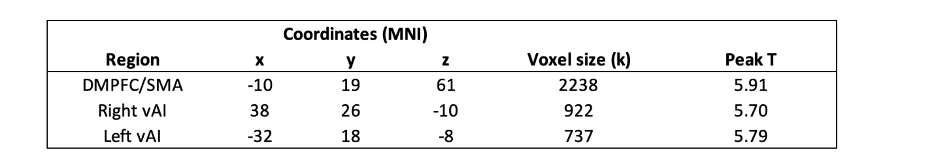


*Supplementary Table 2. Significant clusters found for the Correct>Incorrect univariate contrast map.*


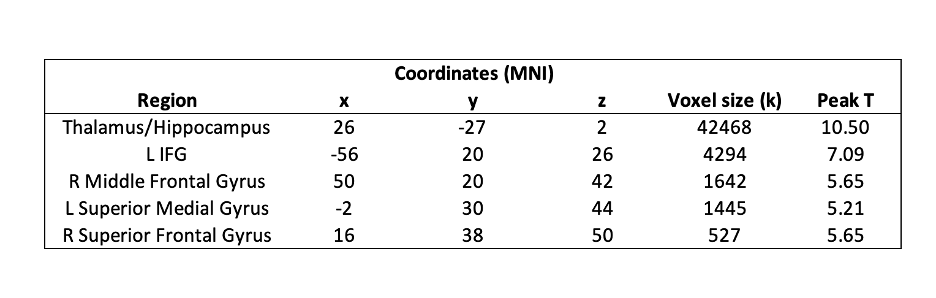


*Supplementary Table 3. Significant clusters found for the Counterpart-Inconsistent>Counterpart-consistent univariate contrast map.*

*
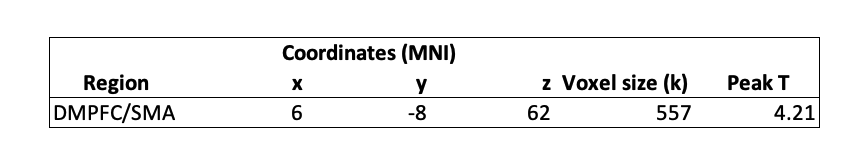
*

*Supplementary Table 4. Significant clusters found for the Self-Inconsistent - Counterpart-Inconsistent univariate contrast map.*

*
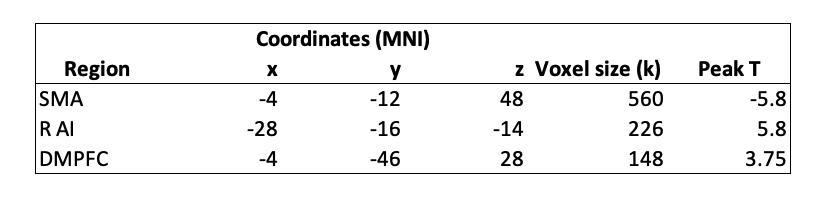
*

*Supplementary Figure 1.* Likeability ratings: Within the self feedback trials, we found a significant valence by consistency interaction (ß(156) = -21.55, *p* < 0.001) such that self-inconsistent feedback was perceived as more negative vs. positive (*t*(39) = 8.91, *p* < 0.001, *d =* 1.41) and self-consistent feedback was perceived as more positive vs. negative (*t*(39) = 12.03, *p* < 0.001, *d* = 1.90) (see Supplementary Figure 1).

*
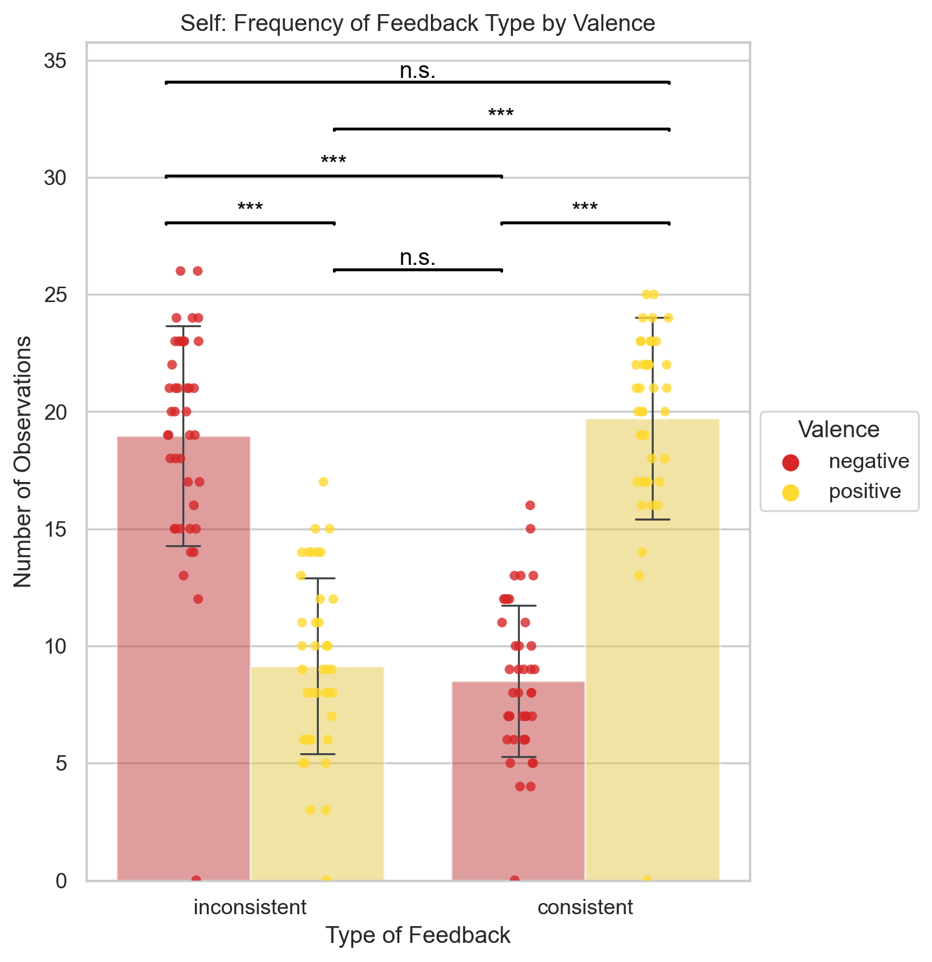
*

*Supplementary Figure 2. Functional hippocampus ROI (in blue) generated by taking the union of the correct>incorrect contrast map and a hippocampus ROI derived from a neurosynth parcellation scheme*


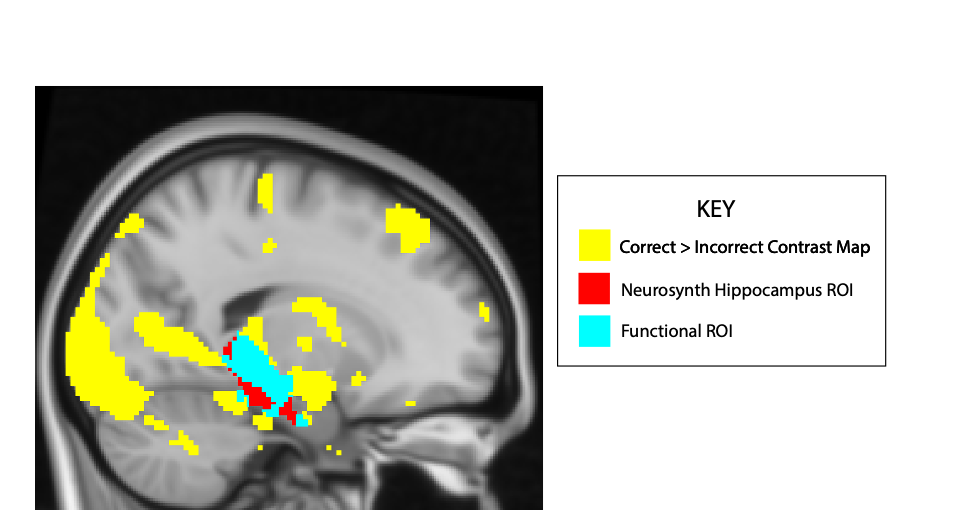


*Supplementary Figure 3. Number of reinstatements in post-self and post-counterpart rest by target (collapsed across consistency) in the DMPFC.*

Within the DMPFC, we found a greater number of reinstatements for self feedback compared to the counterpart feedback during post-self feedback rest (*t*(40) = 3.15, *p* = .003, *d* = 0.49). This pattern was also marginally observed during post-counterpart feedback rest (*t*(40) = 2.06, *p* = .046, *d* = 0.32). Critically, the number of self reinstatements during post-self feedback rest was significantly greater than the number of self reinstatements (*t*(40) = 2.29, *p* = .03, *d* = 0.36) and counterpart reinstatements (*t*(40) = 2.69, *p* = .01, *d* = 0.42) during post-counterpart feedback rest. In other words, the number of DMPFC self-reinstatements was greatest during post-self feedback rest.

*
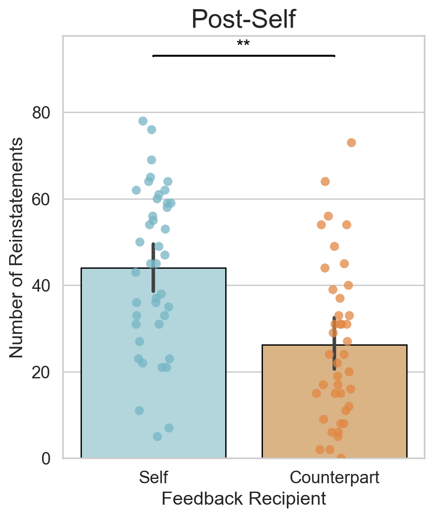
*

*Supplementary Figure 4. Number of reinstatements in post-self and post-counterpart rest by target (collapsed across consistency) in the right AI/IFG.*

Within the right AI/IFG, the self feedback template was reinstated more than the counterpart feedback template during post-self encoding rest (*t*(40) = 2.05, *p* = .047, *d* = 0.32), but not during post-counterpart encoding rest (*t*(40) = 1.56, *p* = .13, *d* = 0.24).


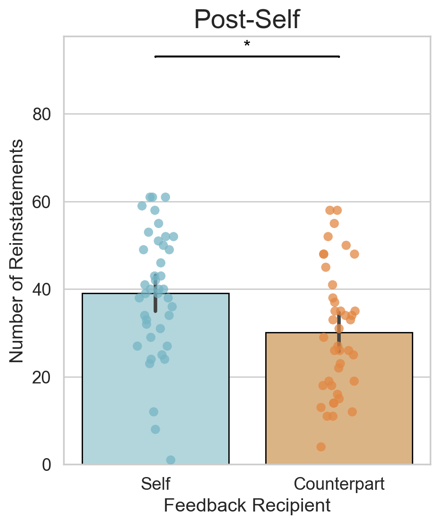


*Supplementary Figure 5. (A) Number of self-inconsistent reinstatements in Right AI/IFG during post-self encoding rest. Inconsistent-self templates were reinstated more than inconsistent-counterpart templates during post-self encoding rest, but not during post-counterpart rest (left graph). During the final rest scan in which all participants had seen all the feedback in the study, self-inconsistent feedback was also not reinstated more than counterpart-inconsistent feedback (right graph). (B) Plot demonstrating that the number of reinstatements of self-inconsistent feedback within Right AI/IFG during post-self encoding rest is positively correlated with subsequently remembered feedback for self-inconsistent items.*

**

*Supplementary Figure 6. (A) Functional hippocampus ROI derived from the Subsequently Remembered>Subsequently Forgotten univariate contrast map. (B) Correlation plot demonstrating that mean levels of activation during encoding of correct items are significantly associated with memory for correct items.*


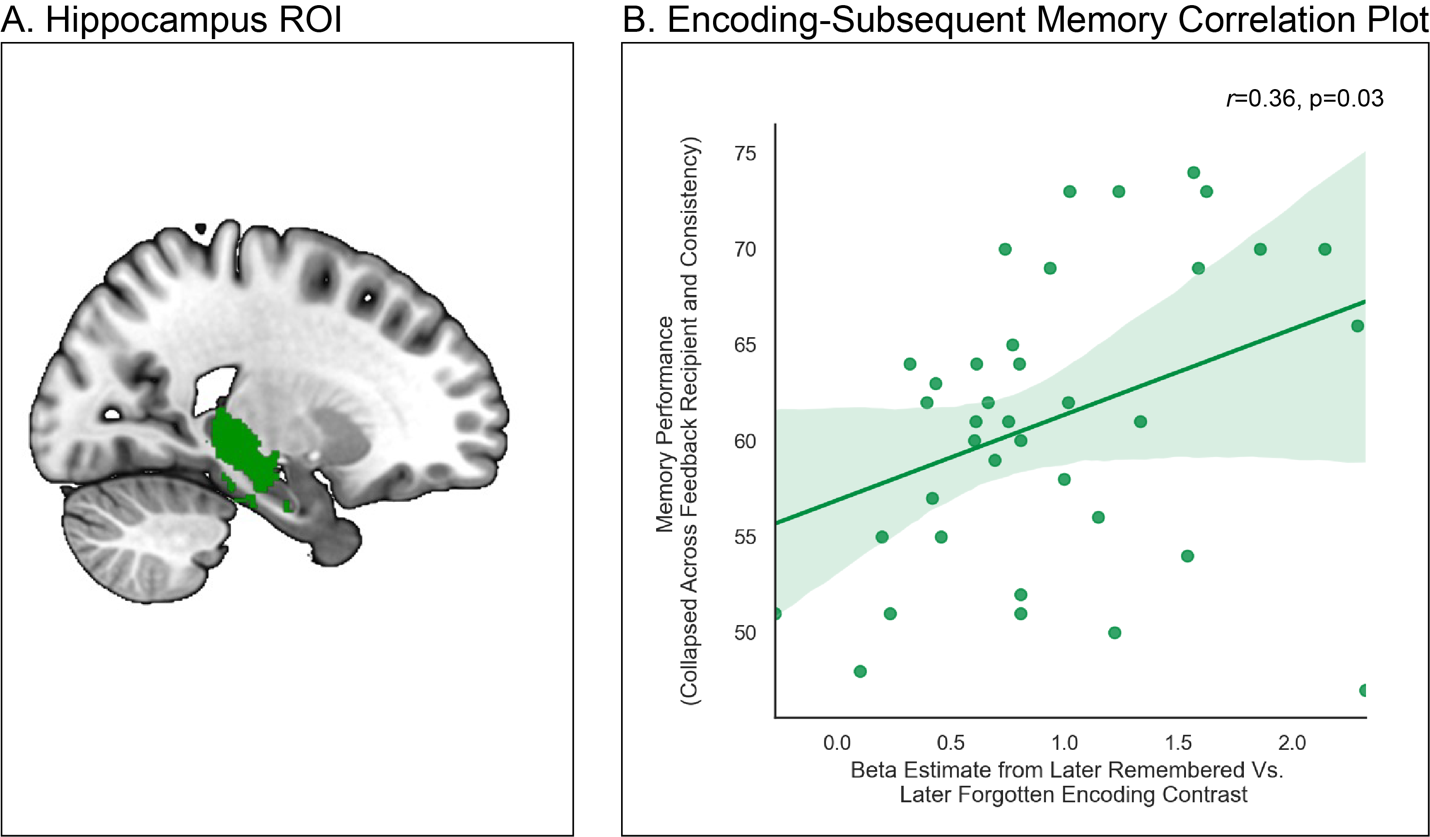

Supplement: Supplementary_Materials_bhae280 [file supplementary_materials_bhae280.docx]
